# Emigration speed and the production of sexuals in colonies of the ant *Temnothorax crassispinus* under high and low levels of disturbance

**DOI:** 10.1007/s00040-015-0447-x

**Published:** 2015-11-19

**Authors:** S. Mitrus

**Affiliations:** Laboratory of Evolution and Animal Ecology, Department of Biosystematics, Opole University, Oleska 22, 45-052 Opole, Poland

**Keywords:** Nest movement, Social insect, Colony size, Energy allocation

## Abstract

A nest relocation is costly for social insects, and involves hazards. Emigrations were studied in *Temnothorax crassispinus* ant colonies, which inhabit ephemeral nest sites, and which frequently change their nests. In a laboratory experiment, ant colonies from one group were forced to change their nest sites 10 times over a ca. 3-month period, whilst colonies from the second group were forced to adopt this practice twice (on the beginning of May, and in the second half of July). Colonies of the ant from both the groups reduced their total emigration duration. However, the duration of the transport phase remained unchanged. In the case of colonies with higher level of disturbance, there was no relation between colony growth rate and energy allocation in sexual individuals, whilst a negative correlation between these parameters was present in group with lower level of disturbance. In colonies with lower level of disturbance, the investment in sexuals was not correlated with the number of workers at the end of the experiment, whereas such a correlation was demonstrated for colonies with higher level of disturbance. The disturbance, and thus necessity of frequent nest relocations, may be perceived by ants as a signal that nest sites are of a lower quality and may contribute to a change in energy allocation.

## Introduction

The life conditions of animals affect their allocation of available energy in growth, reproduction, and the accumulation of reserves, thus affecting their life history parameters (Stearns 1992). Nest site quality is an important factor for social insects. It influences such aspects as food availability, the occurrence of parasites, predation, reproduction, and competition with other colonies (Hölldobler and Wilson 1990; Blüthgen and Feldhaar 2010; Moyano and Feener 2014), which determines life history parameters.

The changing of a nest site is typical for social insects during colony development, and the colonies of some species also change their nest site when they discover a superior one (Hölldobler and Wilson 1990; Dornhaus et al. 2004; McGlynn 2012). However, these emigrations are costly: the sole transport of brood and movement towards a new nest may entail energy costs, but other costs include the risk of a potential predator attack during emigration and the invested duration, which could be used for other purposes, such as food searches (Dornhaus et al. 2004; McGlynn 2012). Probably for the above-mentioned reasons, emigrations to new nest sites in most social insect species are rare, and ant colonies relocate especially after the nest site is destroyed (e.g., Moyano and Feener 2014). Disturbances of nest sites should directly lead to worker and brood loss (Kramer et al. 2013), but also could affect energy allocation: disturbances may affect mortality rate, and thus change the optimal values of life history parameters (cf. Sterans 1992).

In *Temnothorax* ants emigrations may occur several times in one season (Herbers 1989; Herbers and Johnson 2007). In the case of these ants, this is caused by their nesting site: they typically dwell in ephemeral nest sites, and colonies have to emigrate, when the previous nest site is destroyed (Herbers 1989; Sendova-Franks and Franks 1995; Herbers and Johnson 2007; Langridge et al. 2008a). Emigration-related topics were studied in *Temnothorax* ants in numerous laboratory experiments. These studies concerned, e.g., collective decision-making (Langridge et al. 2004; Dornhaus and Franks 2006; Robinson et al. 2011), the ability to choose a nest site (Franks et al. 2003; Dornhaus et al. 2004; Healey and Pratt 2008; Robinson et al. 2009), and specialization among workers (Dornhaus et al. 2008, 2009). It was shown, e.g., that *Temnothorax* ants exhibit division of labour. Colonies of the ants reduced total emigration duration when they repeat the same process. They are able to compare the quality of the nest sites, and chose the best one, as well as correct errors made in choosing nest site. Some of these studies involved forcing ant colonies to emigrate to a new nest site more than once, e.g., to investigate whether the subsequent emigrations might be faster. Typically, such nest movements were forced at 1–3 days intervals (e.g. Dornhaus and Franks 2006; Langridge et al. 2008b), and less frequent were longer intervals between emigrations maintained (e.g., 6, 14, and 20 days; Langridge et al. 2004). Frequent emigrations, e.g., every several hours, can be used in the research into learning processes, but under natural conditions a few such episodes occur per season. In the papers cited above, there was no attempt to correlate emigration issues with life history parameters, and in the case of *Temnothorax* ants, a nest relocation constitutes an important element of their biology (Sendova-Franks and Franks 1995; Langridge et al. 2008a). In this paper I conducted an experimental study of emigrations in *T.* *crassispinus* ant colonies. The aim of this paper was to investigate how disturbance (caused frequent nest relocations, versus infrequent movements) affect the duration of ant emigration to a new nest and whether they influence life history parameters, and primarily investment in sex individuals, but also colony growth.

## Materials and methods

### Study organisms

The cavity-dwelling ant *Temnothorax crassispinus* is present throughout Western and Central Europe (Seifert 2007; Czechowski et al. 2012) and it is a species widely distributed in Poland, known mostly from sites in coniferous forests (Czechowski et al. 2012). They are among the most widely distributed and most common ant species. Colonies of the *Temnothorax* species are small, ranging from a few dozen to several hundreds of workers (Seifert 2007; Czechowski et al. 2012) and are usually monogynous (have one queen) (Heinze and Buschinger 1988; Seifert 2007; Seifert 2008; Czechowski et al. 2012). The *T. crassispinus* ant colonies inhabit mostly cavities in acorns and in sticks in the leaf litter layer. Good nest sites are probably limited resources and colonies may be frequently forced to find a new nest site (Herbers 1989; Herbers and Johnson 2007).

### Laboratory experiment

**Fig. 1:**
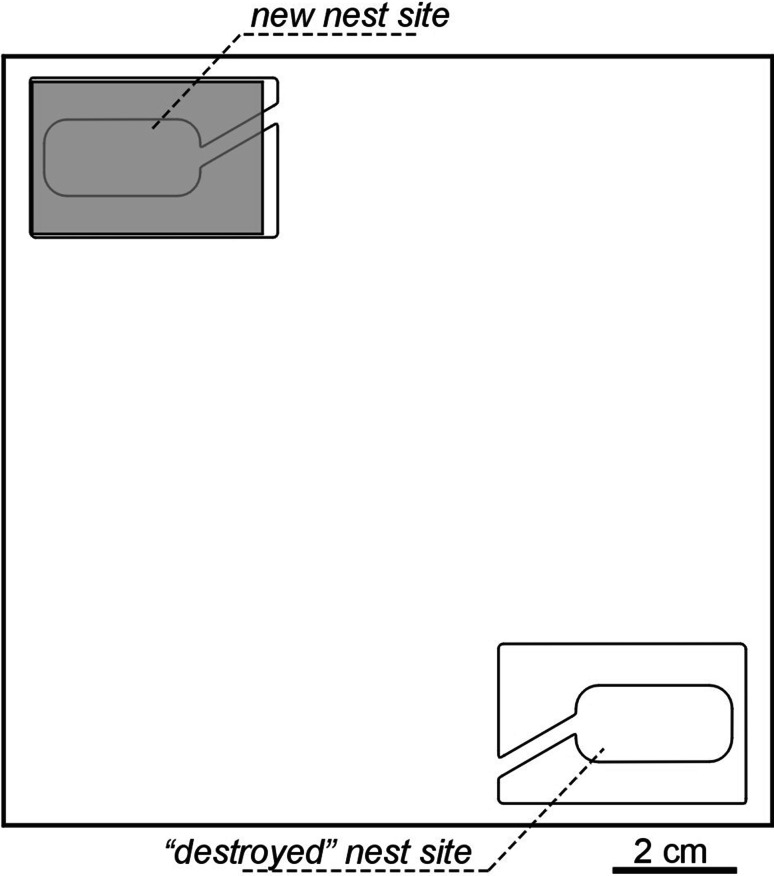
Diagrammatic representation of the relative positions of the old and new nest sites of the ant *Temnothorax crassispinus* on the Petri dish (10.2 cm × 10.2 cm × 1.9 cm), when experimental emigration were forced. The new nest sites were placed on the Petri dish, and then the old nests were “destroyed” (=opened by removing its top glass)

On 23 April 2013, near Opole, I collected sticks and acorns housing *T. crassispinus* ant colonies. The nest sites were transported to a laboratory, where they were carefully opened, the ants captured with an aspirator and counted. I collected 70 nests with ant colonies (=nest cavities with ants and broods at different developmental stages) containing 19–149 workers (median: 68.5, quartiles: 41.25–98) and 0–4 queens; 42 of the nests contained one queen. I also found 20 nest sites containing only workers, without brood (1–14 workers, median: 4), and one site with a lone queen. In the experiment I used 40 colonies with one queen and 25–149 workers (average: 77.4, SD = 35.0). They were transferred to square Petri dishes (10.2 cm × 10.2 cm × 1.9 cm) with a thin plaster base and an artificial nest chamber placed on top. Each nest chamber was made of a 3 mm thick, fittingly carved plexiglass frame (see: Fig. 1), sandwiched between two 1/2 microscope slides. A piece of cardboard providing the base for the nest was placed between the bottom microscope slide and the plexiglass form. The whole nest was coated with a piece of red translucent filter from above. The upper microscope slide was fastened to the plexiglass frame from one side only, making it possible to open the nest site (to induce emigrations, see below).

The Petri dishes with colonies were kept in a Pol-Eko ST 1 thermostatic cabinet (manufactured by POL-EKO Aparatura sp.j.) maintaining a daily cycle of 12:12 h, 20 and 10 °C, starting April 23 to June 24, and 14:10 h, 27 and 17 °C, for the second part of the experiment. This set up follows the conditions used in previous experiments mimicking spring and summer conditions for *T. nylanderi* and *T. crassispinus* ants (Foitzik et al. 2003; Mitrus 2015). The ants were fed twice a week with frozen *Drosophila hydei* fruit flies and honey. The colonies were randomly divided into two groups; the groups did not differ in initial number of workers (*t* Student test, *t* = 0.16, *df* = 38, *p* = 0.87). During experimental procedure, I placed the second nest site on the opposite side of the Petri dish (distance between entrances ca. 11 cm, Fig. 1, nest sites were always in the same locations), and then “destroyed” the inhabited nest site (=opened by removing its top glass). This procedure started 2 h after the “day” began at the thermostatic cabinet; food and water was removed from the Petri dishes before the procedure. Location of the Petri dishes in the thermostatic cabinet was randomised; however, nest site was always in the same direction.

At the beginning of the experiment, on 7–10 May 2013, the “old” nests of all colonies from both the groups—one with higher level of disturbance (=high disturbance), and the second with lower level of disturbance (=low disturbance)—were destroyed. Every week since 22 May, I opened nests from the high disturbance group and forced the ant colonies to move to a new nest site. At the same time colonies from low disturbance group were also offered “new” nests; the “new” nest sites were then left for 24 h and then—if not inhabited—removed from the Petri dishes. On 23–26 July—during the last emigration for the high disturbance group—all nests, from both groups, were opened. Ultimately, during the experiment, colonies from the group with higher level of disturbance were forced to emigrate 10 times, and colonies from the group with lower level of disturbance—two times. The order, in which colonies were forced to emigrate, was randomized. The plexiglass frame and microscope slides from “old” nests (and “new” ones from the low disturbance group, when not inhabited) used to construct new nest sites, were cleaned with water and alcohol after each emigration to remove any pheromones. New pieces of cardboard were used to construct nest sites each time.

Emigrations to new nesting sites, of 15 randomly chosen colonies from the low disturbance and 15 from the high disturbance groups, were recorded with a digital video camera: on 7–10 May (the first emigration), on 3–6 June (the 4th emigration for the high disturbance group), and on 23–26 July (the last emigration for colonies from the high disturbance group; the second emigration for colonies from the low disturbance group). At the same time, the behaviour of ants in two Petri dishes was recorded. The camera was positioned above so that the camera’s field of view encompassed the two dishes. During recording, the Petri dishes were outside of the thermostatic cabinet. However, at that time in the laboratory the temperature was similar to the temperature in the cabinet: on 7–10 May 20.0–22.2 °C, on 3–6 June 18.2–19.0 °C, and on 23–26 July 26.0–27.0 °C.

All the videos were analysed twice. Similar to other studies (e.g., Stroeymeyt et al. 2010) I determined: (1) the discovery phase duration (interval from the time the “old” nest was destroyed to the time the new nest was first entered by a worker); (2) the assessment phase duration (interval from the time the new nest was discovered to the time the first brood or worker was carried into the new nest site); and (3) the transport phase duration (interval from the time the first brood or worker was carried into the new nest site to the time the last brood was carried into the nest).

On 29 July, I counted the final number of workers in each colony, and the number of sexual individuals produced by each colony. Then, I estimated relative cost of production of sexual individuals (investment into sexuals): in order to estimate the cost I used number of produced males and females, and adopted literature data for the ant *T. nylanderi* (a sibling species of *T. crassispinus*), according to which the dry mass and hence the cost of a young queen being produced, is 3.02 times higher than that of a male, and dry mass of workers does not differ from male dry mass (Foitzik and Heinze 2000; Foitzik et al. 2003). Thus, relative cost of production of sexuals by colony was calculated as: number of produced males + 3.02 × number of produced females.

### Statistical analysis

Two colonies (both from the low disturbance group) were excluded from the analyses: in one the queen died in May and a number of workers decreased; in the second colony the number of workers decreased considerably (from 109 to 38 workers during the experiment, although the queen was still alive), but the reason for the change is not known.

Statistical analyses were carried out using the software package Statistica, ver. 10 (StatSoft Inc. 2011). *U* Mann–Whitney tests were used to compare the discovery phase duration, the assessment phase duration and the transport phase duration between colonies from the low and high disturbance groups. I used the Wilcoxon signed rank test to compare duration of the phases between emigration on 7–10 May, and on 23–26 July comparing the same colonies. Spearman or Pearson correlations were used to assess the relationship between colony growth rates, allocation of energy in sexual individuals, and number of workers in colonies. To analyze whether investment into sexual individuals (cost of production of sexuals) was associated with treatment, I conducted an analysis of covariance (ANCOVA) with growth rate as a continuous predictor. I used the non-parametric statistics when data sets were significantly different from normal distribution, even after transformation. When multiple tests were performed, I corrected the *α*-values according to the Bonferroni approach (Sokal and Rohlf 1995)—thus, the corrected significance level was *α′* = 0.05/*N*, where *N* = number of the multiple comparisons. All probability values shown are two-tailed.

## Results

All colonies from the destroyed nests moved to the new ones offered. In the group with lower level of disturbance, where the old nest was left intact, none of the ant colonies changed their nest for a new one, although the new nest sites were sought out and penetrated by workers. Ants from the low disturbance group did not move to new nests even when fungus appeared in some nests (all from the low disturbance group) in the second half of the experiment.

**Fig. 2:**
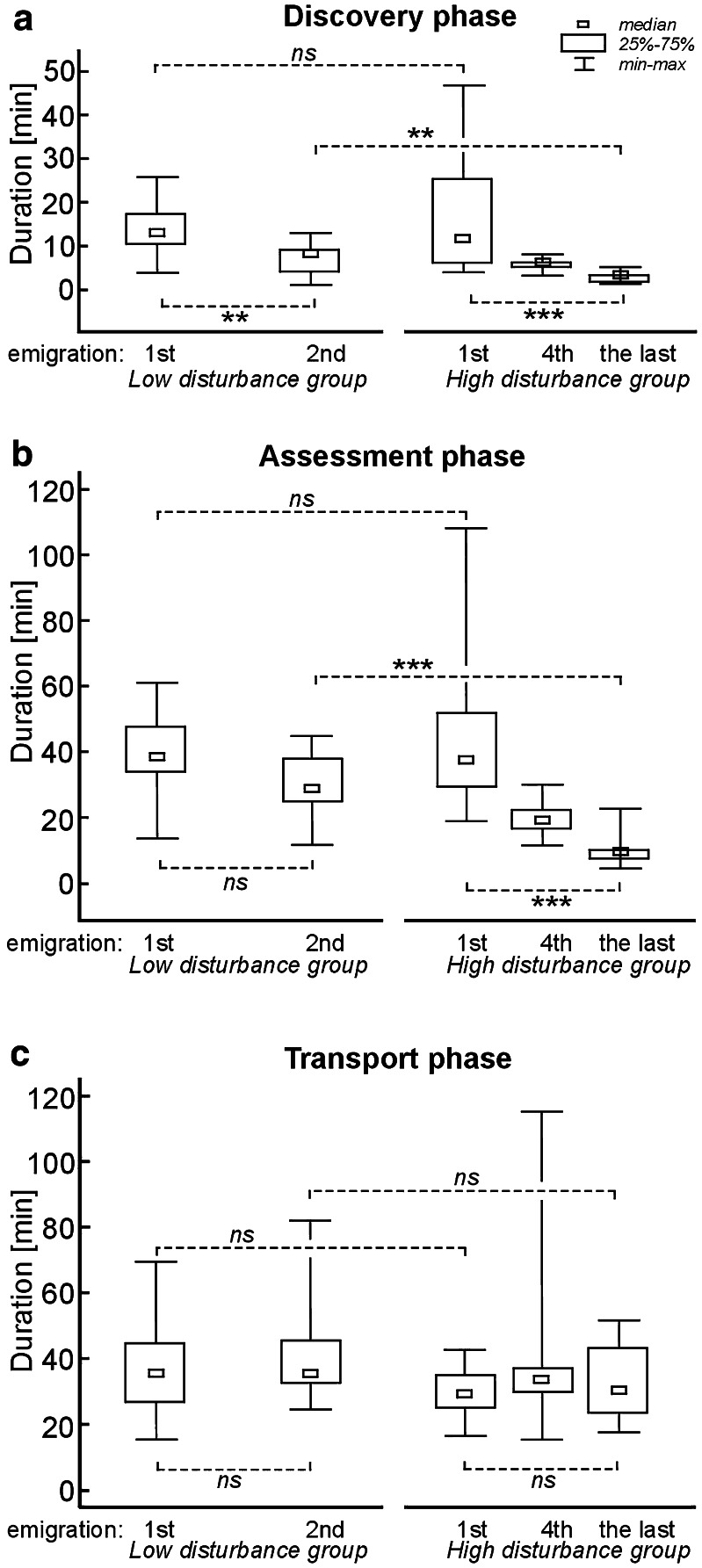
Results for the emigration phases of ant colonies of *T. crassispinus*: **a** discovery duration; **b** assessment duration, and **c** transport duration, for colonies from the group with lower level of disturbance (low disturbance) [*N* = 15] and with higher level of disturbance group (high disturbance) [*N* = 15; or *N* = 14 for assessment duration and transport duration during 1st emigration, and for all the emigration phases during 4th emigration]. Colonies from the low disturbance group were forced to emigrate twice: on 7–10 May, and on 23–26 July; colonies from high disturbance group were forced to emigrate 10 times: the first being on 7–10 May, then every week since 22 May, with the last time being on 23–26 July (the 4th emigrations were on 5–6 June). **p < 0.01, ***p < 0.001, *ns* = not significant; in *U* Mann–Whitney test or Wilcoxon signed rank test, to compare the durations between low and high disturbance groups, and to compare the durations between emigration on 7–10 May, and on 23–26 July when comparing the same colonies, respectively

There were no differences between colonies from the low disturbance group and the high disturbance group in the durations of particular emigration phases, at the beginning of the experiment (*U* Mann–Whitney tests, *U* from 70.5 to 100.5, *p* from 0.21 to 0.97). Such differences were shown at the end of the experiment, where durations of discovery phase and assessment phase were shorter in the high disturbance colonies (*U* = 35.5, *N*_1_ = 15, *N*_2_ = 15, *p* = 0.0015 and *U* = 14, *N*_1_ = 15, *N*_2_ = 15, *p* < 0.0001). No differences were found in the duration of transport phase between the low and high disturbance colonies during the last emigration (*U* = 74.5, *N*_1_ = 15, *N*_2_ = 15, *p* = 0.12; Fig. 2; Table 1).

**Table 1:** Duration of the emigration phases of *Temnothorax crassispinus* ant colonies when they were forced to emigrate

	Low disturbance group			High disturbance group			
	Median min–max			Median min–max			
	1st emigration [7–10 May]			1st emigration [7–10 May]			
Discovery phase duration	13	4–26	*N* = 15	12	4–47	*N* = 15	*ns*
Assessment phase duration	26	9–48	*N* = 15	24	9–73	*N* = 14	*ns*
Transport phase duration	36	16–70	*N* = 14	29.5	17–43	*N* = 14	*ns*
				4th emigration [3–6 June]			
Discovery phase duration				6	3–8	*N* = 14	
Assessment phase duration				13.5	9–23	*N* = 14	
Transport phase duration				34	16–115	*N* = 14	
	2nd emigration [23–26 July]			the last (10th) emigration [23–26 July]			
Discovery phase duration	8	1–13	*N* = 15	3	1–5	*N* = 15	**
Assessment phase duration	24	7–43	*N* = 15	7	3–20	*N* = 15	***
Transport phase duration	36	25–82	*N* = 15	31	18–52	*N* = 15	*ns*

Colonies from the group with lower level of disturbance group (low disturbance) were forced to emigrate twice; colonies from group with higher level of disturbance group (high disturbance) were forced to emigrate 10 times. Median, min–max [in min] are shown. Differences in number (*N*) of analysed colonies during emigration are the result of technical problems with the camera

** *p* < 0.01, *** *p* < 0.001, *ns* not significant, in *U* Mann–Whitney test

When comparing the same ant colonies at the beginning and end of the experiment, the discovery phase duration was significantly shorter both for the low and high disturbance colonies (Wilcoxon signed rank test, *T* = 6.0, *N* = 15, *p* = 0.0022 and *T* = 0.0, *N* = 15, *p* < 0.001; Fig. 2). In the case of colonies from the high disturbance group, but not the low disturbance group, the assessment was also reduced (*T* = 0.0, *N* = 14, *p* < 0.001 and *T* = 38.5, *N* = 14, *p* = 0.37, respectively). The duration of the transport of brood and workers to a new nest remained unchanged, both in colonies from the low and high disturbance group (*T* = 45.5, *N* = 14, *p* = 0.66 and *T* = 35.0, *N* = 13, *p* = 0.46; Fig. 2).

There was no correlation between the number of workers and the duration of particular emigration phases at the beginning of the experiment, either in the low and high disturbance colonies (*R*_s_ from –0.54 to 0.14, *p* from 0.046 to 0.95; there is no significant correlation for *p* = 0.046 taking into consideration the Bonferroni correction: as *α′* = 0.05/*N*, and *N* = 6, thus *α′* = 0.0083). Similarly, no correlation was found in the case of results obtained at the end of the experiment (*R*_*s*_ = 0.64, *N* = 15, *p* = 0.0089 for the number of workers and transport phase duration in the high disturbance colonies, however, *α′* = 0.0083 due to there being six comparisons; for other emigration phases: *R*_s_ from –0.32 to 0.37, *p* from 0.20 to 0.81).

Colonies from the high disturbance group produced 0–41 sexual individuals (median 12.5, *N* = 20), and those from the low disturbance group produced 0–67 (median 7, *N* = 18); four colonies (two from the high disturbance and two from the low disturbance group) produced no sexual individuals. Most colonies produced only males (17 out of 20 and 13 out of 18 colonies, in the high and low disturbance group, respectively). Only four colonies produced queens: one colony from high disturbance group produced 10 females and 5 males, three colonies from low disturbance group 17/45, 6/31, and 9/16 females/males, respectively.

**Fig. 3:**
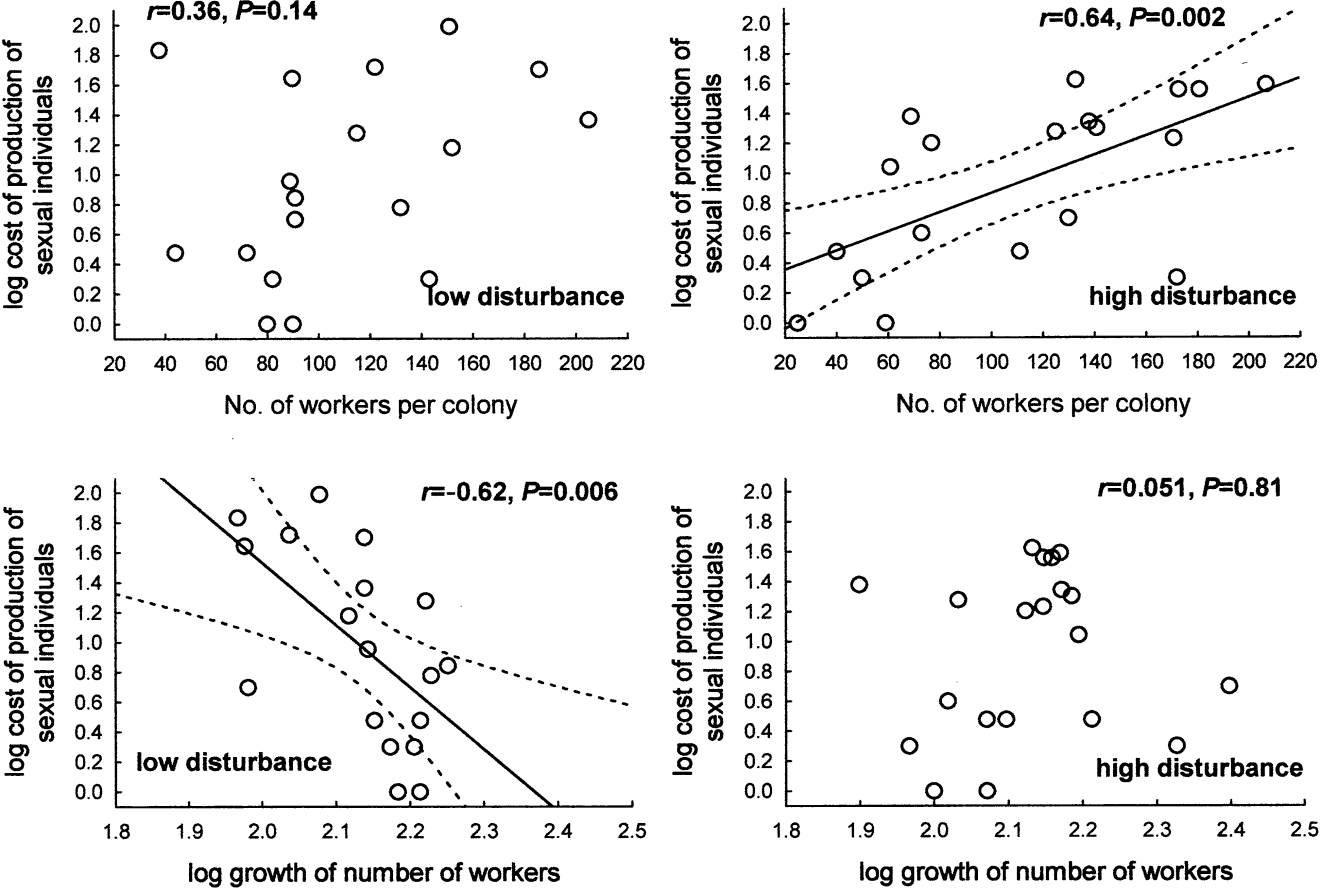
Cost of production of sexual individuals in ant colonies of *Temnothorax crassispinus* kept in laboratory conditions in relation to growth rate and the final number of workers. Colonies from the group with lower level of disturbance (low disturbance) [*N* = 18] were forced to emigrate twice; colonies with higher level of disturbance group (high disturbance) [*N* = 20] were forced to emigrate 10 times

The groups did not differ in the investment into sexuals (=cost of production of sexual individuals; ANCOVA, *F*_1,35_ = 0.10, *p* = 0.75, growth rate used as continuous predictor). The change in the number of workers during the experiment was negatively correlated with cost of production of sexual individuals in colonies from the low disturbance group (*r* = –0.62, *N* = 18, *p* = 0.006, and *R*_s_ = –0.59, *N* = 18, *p* = 0.0092, denoting total investment in sexuals and per-capita investment in sexuals, respectively). No such relationship was found for colonies from the high disturbance group (*r* = 0.051, *N* = 20, *p* = 0.81, and *R*_s_ = –0.01, *N* = 20, *p* = 0.97, Fig. 3). On the other hand, the number of workers at the end of the experiment was not correlated with the investment in sexual individuals in colonies from the low disturbance group (*r* = 0.36, *N* = 18, *p* = 0.14), whereas such a positive correlation was shown for colonies from the high disturbance group (*r* = 0.64, *N* = 20, *p* = 0.002; Fig. 3). I found no correlation between the number of workers at the end of the experiment and per-capita investment in sexual individuals (*R*_s_ = 0.25, *N* = 18, *p* = 0.31 and *R*_s_ = 0.42, *N* = 20, *p* = 0.068, for colonies from the low and high disturbance group, respectively).

## Discussion

Previous research into emigrations in *Temnothorax* ants was mostly conducted in the form of short-term experiments and was devoid of any attempt to associate costs with life history parameters. In this experiment, I tried to investigate whether any differences can be noticed in the allocation of energy in sexual individuals between colonies from with higher and lower level of disturbance. Distance between old and new nest sites in the study was ca. 11 cm only, and mean foraging distance at *T. nylanderi* was 52 cm (Fokuhl et al. 2012). Thus, I think that observed effects are rather effects lower/higher level of disturbance of nest sites, not caused directly by the costs related to movements to new nest sites. At the same time, the frequent “damage” to nest sites (and the resultant necessity of nest relocations) may be perceived by ants as a signal of a lower quality of nest sites/area where they live, and may thus also co-determine a change in energy allocation. Strätz and Heinze (2004) demonstrated that a male-biased sex ratio among sexual individuals is characteristic of ants dwelling in inferior nest sites. In this study, a substantial majority of colonies produced only males, and it was impossible to compare energy allocation into the production of individuals representing each sex. The colonies were kept in Petri dishes for a considerable duration during the experiment, and it is possible that the conditions were not optimal for ant development.

In ants per-capita productivity often declines in larger colonies, but *T. crassispinus* did not show a decrease in per-capita productivity with increasing colony size (Kramer et al. 2013; Mitrus 2015). Neither in this experiment did I find any correlation of per-capita productivity (for sexual individuals only) with colony size. The limited available resources need to be divided between growth and reproduction. In the case of colonies from the group with lower level of disturbance, as was expected, I found a negative correlation between colony growth rate and energy allocation in sexuals. There was no such relationship in colonies from the group with higher level of disturbance: however, it is possible that under the level of disturbance used in the experiment, growth rate and/or reproduction were not optimal. Differences in energy allocation may affect energy reserves, thus exerting an impact on, e.g., the survival of the colony in the forthcoming periods (including winter) and/or on the possibility of development and reproduction in the subsequent season. This, however, was not the subject of the presented paper, and—given that the culture conditions were probably non-optimal—research of this kind would have to be conducted either in natural conditions or in laboratory conditions more favourable to ant development.

High variance in investment in growth and sexual reproduction could be caused by the ant colonies ‘personalities’: ant colonies differ reputably in their behaviour, and the behaviour could have consequences for life history parameters (cf. Bengston and Dornhaus 2014). I have no data on natural variation in investment in sexuals between colonies. Thus it is impossible to distinguish natural variation in investment in sexuals, from the change in investment, caused by level of disturbance in the experiment.

Finding a new nest site and emigration to it may be risky and involve considerable duration and energy input (Dornhaus et al. 2004; McGlynn 2012). None of the colonies from the low disturbance group changed their nest site in the presented experiment, unless their previous nest site was experimentally destroyed. This is consistent with previous suggestions (Dornhaus et al. 2004) that the cost and/or risk associated with emigrations in natural conditions may be quite substantial.

The duration put into finding a new nest and the durations of analysed emigration phases determined in this experiment, are generally compatible with previous findings concerning *Temnothorax* ants (cf. e.g. Langridge et al. 2004; Langridge et al. 2008a). Differences may ensue from the conditions prevailing during experiments, such as distances between nests and ambient temperatures. It was demonstrated in the previous studies that ant colonies moved to a new nest more quickly during successive emigrations (e.g. Langridge et al. 2004; Langridge et al. 2008a). In this study I also noted that discovery and assessment phase durations grew shorter. In the case of colonies from the low disturbance group—in which emigrations were forced at approximately 11 week intervals—the duration spent on discovering the new nest was decreased. However, in the study by Langridge and co-workers (2004), it was shown that emigration phase duration was not reduced if the interval between emigrations was longer than 6 days. Disparities between these findings rather could not be due to the temperature in a laboratory during this experiment, amounting to 20.0–22.2 °C during the first emigration and 26.0–27.0 °C on 23–26 July, which may have affected the activity of workers. I think it is unlikely that temperature caused the observed changes, as I noted no differences in either the low or the high disturbance group in terms of the duration of transport between nests. What more, in the high disturbance group, the reduction in both discovery and assessment phases duration were already present on the 4th transport (June 3–6), which happened before the temperature increase (June 24th) (cf. Fig. 2). This indicates that temperature differences during these emigrations are not a key factor determining the dissimilarities between the analysed durations of individual emigration episodes. Transport duration is likely to be minimised as it is probably the most hazardous emigration phase, e.g., queen, more workers, and transported brood are given potential predator attacks (e.g. Franks et al. 2003; Langridge et al. 2004). Results of present experiment suggest that in ant colonies reduction of total durations over successive emigration could be effective also with longer periods between emigrations. However, it is impossible to say now if the disparities with previous studies could be seasonal effect (cf. Stroeymeyt et al. 2014) or different studied species: in the study Langridge and co-workers (2004) *T. albipennis* ant colonies were used. I found no correlation between the durations of emigration phases and the number of workers. Although this is consistent with other papers (e.g. Dornhaus and Franks 2006), it remains puzzling because the probability of a new nest site being accidentally discovered should be higher if the number of workers is larger. Perhaps, as suggested by Dornhaus and Franks (2006), this is caused by experimental conditions, and such a relationship could be shown in a more diverse environment and/or with new nest sites located at larger distances. In this study, I noted only a correlation between the number of workers and transport phase duration at the end of the experiment in the high disturbance group. This result is difficult to interpret. However, transport phase duration may depend on, e.g., the number of larvae and pupae that must be carried to a new nest, whereas the number of eggs, larvae and pupae was not determined in this study.

Issues concerning energy allocation into growth and reproduction in social insects still remain an unresolved problem of sociobiology (cf. Poitrineau et al. 2009). Ants of the genus *Temnothorax* are a classic study subject in social insect research, and frequent nest-site changeovers are a typical element of their ecology. This study demonstrates that colonies of ant *T. crassispinus* reduced total emigration duration, even with longer periods between emigrations, and that the frequency of emigrations affects energy allocation.
